# Storm events influence the transport of macroplastics in urban streams

**DOI:** 10.1002/wer.70083

**Published:** 2025-06-01

**Authors:** Bailey A. Schwenk, Elizabeth M. Kazmierczak, Fritz Petersen, Jacob Haney, Xia Zhu, Shan Zuidema, Emily K. Lever, Richard B. Lammers, Wilfred M. Wollheim, Chelsea M. Rochman, Timothy J. Hoellein

**Affiliations:** ^1^ Department of Biology Loyola University Chicago Chicago Illinois USA; ^2^ Department of Ecology and Evolutionary Biology University of Toronto Toronto Ontario Canada; ^3^ Earth Systems Research Center University of New Hampshire Durham NH USA; ^4^ College of Life Sciences and Agriculture University of New Hampshire Durham New Hampshire USA

**Keywords:** camera, macroplastic, storms, stormwater, surface water, urban rivers, water pollution

## Abstract

Plastic litter is a globally pervasive pollutant. Storms are likely key drivers of plastic transport to oceans, but plastic transport during rising and falling limbs of storm hydrographs is rarely measured. Measurements of plastic movement throughout individual storms will improve watershed models of plastic dynamics. We used cameras to quantify macroplastic movement (i.e., particles > 5 mm) in rivers before, during, and after individual storms (N = 18) at 10 sites within three North American watersheds. Most storms showed no difference in macroplastic transport between rising and falling hydrograph limbs or evidence of hysteresis (transport rate range = 0–236 items/30 min). Total macroplastic exported during storm events was positively related to storm magnitude and was greatest at more urban sites. Thus, macroplastic transport during storms was driven by storm size and land use. The quantitative relationships between macroplastic movement and hydrology will improve discharge‐weighted calculations of macroplastic transport which can benefit modeling, monitoring, and mitigation efforts.

**Practitioner Points:**

- Macroplastic particles (i.e, > 5 mm) are both retained in urban streams (e.g., in debris dams), and move downstream during baseflow and stormflow conditions
- Storm flows are key periods of macroplastic transport: transport rates are higher on both rising and falling limbs of storm hydrographs relative to baseflow.
- The amount of macroplastics moving during storm flows is positively related to storm intensity.
- The predictive relationships generated between storm flow and macroplastic transport will improve estimates of annual export, and policies for macroplastic pollution reduction.

## Graphical abstract

We found higher rates of macroplastic (particles > 5 mm) transport rate in urban streams during storms relative to baseflow. Transport was related to storm size. Results will improve predictive models of macroplastic transport and inform policies to reduce macroplastic pollution.

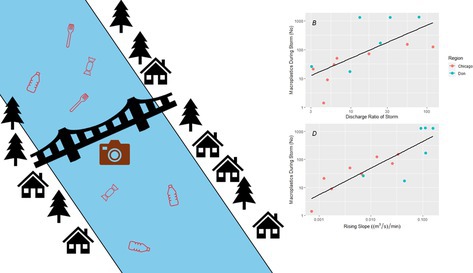

## INTRODUCTION

Plastic litter is a ubiquitous pollutant in ecosystems worldwide. The production of new plastic and the generation of plastic waste has accelerated annually since widespread production began in the mid‐1900s (Geyer et al., 2017). Approximately 6300 million metric tons (Mt) of plastic waste was generated from 1950 to 2015 (Geyer et al., 2017). Of the plastic waste generated, approximately 9% was recycled, 12% was incinerated, and 79% entered landfills or the environment (Geyer et al., 2017). Plastic litter is well documented across all environments including rivers, the deep ocean, and isolated mountaintops (MacLeod et al., 2021).

Rivers transport litter to oceans and lakes, which are a major sink for plastic pollution (Eriksen et al., 2023; Martin et al., 2022; Zhu et al., 2024). However, rivers also retain, transform, and control the timing of plastic export downstream (Hoellein & Rochman, 2021; Liro et al., 2020; van Emmerik & Schwarz, 2020). While a considerable body of research has focused on the export of plastic from rivers to oceans and lakes, analysis of plastic movement within rivers is emerging relatively recently (Windsor et al., 2019; van Emmerik et al., 2022). This quickly growing field of study benefits from building on the fundamental understanding of particle transport dynamics in lotic ecosystems (Hoellein & Rochman, 2021; Roebroek et al., 2024).

The study of particle transport in rivers is a long‐standing field of research and includes a particular emphasis on variation in discharge as a driving factor. Stormwater carries materials such as sediments, coarse particulate organic matter, and anthropogenic litter into streams (Lee & Bang, 2000). The initial rush of stormwater into a stream can carry a higher concentration of particles than the later stages of a storm flow event. The ‘first flush’ is defined as the pollution load transported by the first 20% of stormwater runoff volume per storm event (Deletic, 1998; Lee et al., 2002). The role of the first flush and stormwater in affecting particle loads over long time periods has been well‐studied for naturally occurring particles and particulate pollutants (Do et al., 2023; Morgan et al., 2017; Stenstrom & Kayhanian, 2005) but is rarely applied to studies on transport of macroplastic litter (i.e., particles > 5 mm) (Cowger et al., 2022).

An emerging field of study has assessed plastic pollution in streams relative to changing discharge, comparing plastic abundance between high and low flows, or before and after storm events. Van Emmerik et al. (2019) found macroplastic transport was higher at high flow relative to lower flow in the Seine River (Paris, France). During a major flood in Meuse (Netherlands), van Emmerik et al. (2023) found greater plastic transport rates at peak flood discharge relative to baseflow conditions. Similarly, microplastics (i.e., particles < 5 mm) are re‐suspended and exported from streams during storm events (Berg et al., 2024; Gündoğdu et al., 2018; Hurley et al., 2018). The composite results suggest that plastic concentrations increase at higher discharge due to terrestrial input and remobilization of particles retained in rivers. Yet, assessments of plastic transport rates across the rising and falling limbs of individual storm events are needed to determine if the first flush pattern occurs for macroplastic pollution and to generate predictive models of storms impacts on plastic export.

Our goal was to quantify the amount and types of macroplastics moving through rivers before, during, and after individual storm events in urban streams. We used a video‐based approach to capture macroplastic flux during 12 events at 10 stream sites with varying watershed sizes across three different watersheds. We expected storms would add macroplastic to the rivers and remobilize the macroplastic already retained within the rivers. Thus, we predicted that macroplastic transport rate (i.e., flux in units of number/time) would be significantly greater during storm events relative to before and after. We expected macroplastic to follow the first flush pattern, which would be evident from a higher flux of particles at the rising limb discharge, relative to a lower concentration on the falling limb at the same discharge (i.e., clockwise hysteresis). We predicted that the flux of plastic at downstream sites would be significantly higher than at upstream sites due to the increasing number of point and non‐point sources of plastic with increasing drainage area. Finally, we predicted that the macroplastic items found at higher discharge during storm events would be larger (i.e., higher discharge can move larger particles), and more likely to include hygiene products (i.e., potential items from combined sewer overflows) than plastics at low discharge.

## METHODS

### Study sites

**FIGURE 1:**
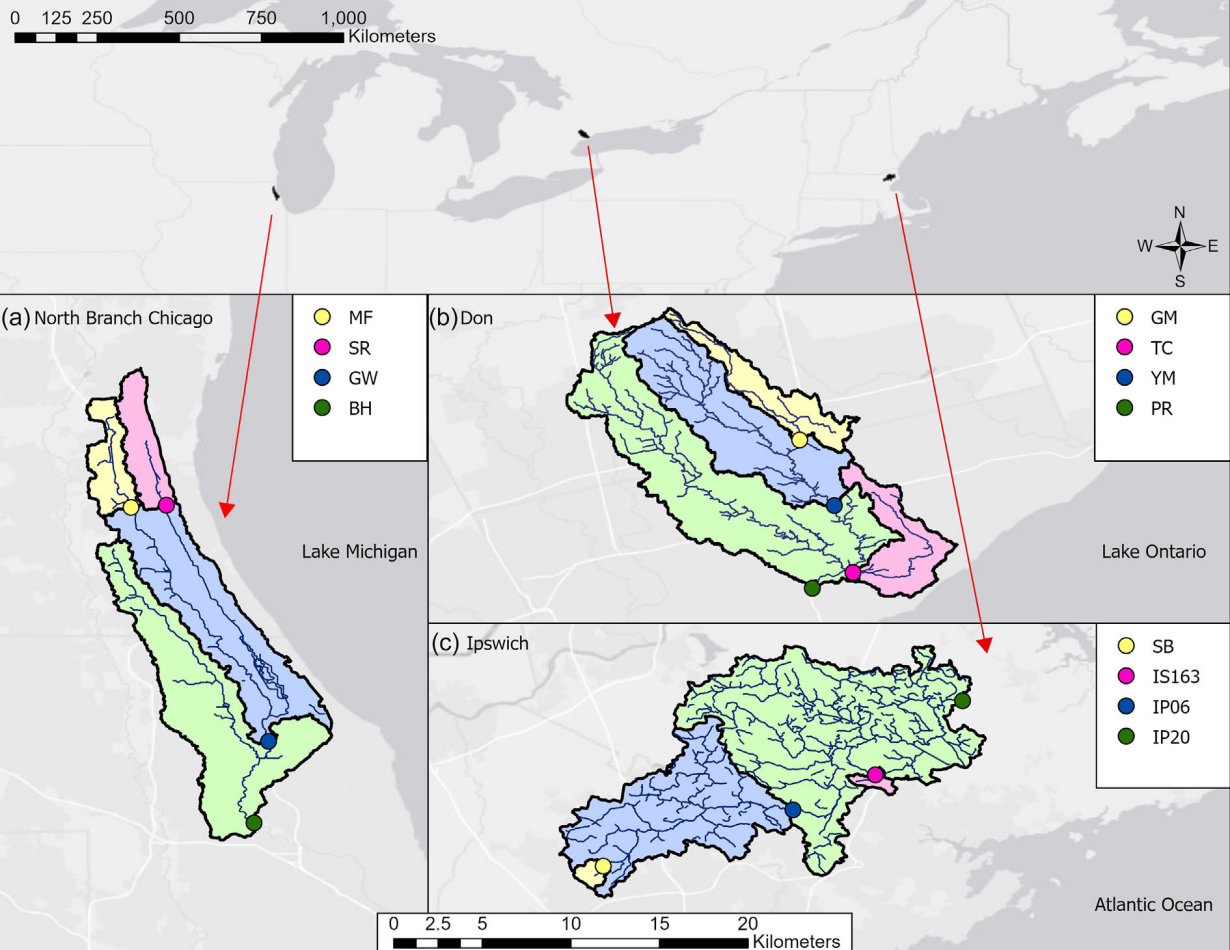
This study was conducted in three watersheds in North America (top panel): North branch Chicago River (A), Don River (B), and Ipswich River (C). Each watershed has a site downstream (green), one site mid‐watershed (blue), and two upstream sites (yellow and purple).

Our study design encompassed a total of 10 sites spread across three watersheds: the North Branch Chicago River (Illinois, USA), Ipswich River (Massachusetts, USA), and Don River (Ontario, Canada). Each river had four sites: two headwater streams, one mid‐river site, and one site near the watershed outlet (Figure 1). All but one study site had a bridge to deploy equipment, and all sites were located near an automated discharge gauge. These sites were selected as part of a larger study designed to measure the influence of storms on the abundance and transport of micro‐ and macroplastic at the watershed scale (Haney et al., In press; Schwenk, 2024). All watersheds have perennial flow, four distinct seasons, and gradients of urban land use. The greatest urban land use was in the Don River sites (77–91%), followed by the Chicago River sites (64–81%), and then the Ipswich River sites (39–88%) (Table S1; Figure S1) (Schwenk, 2024).

### Camera‐based data collection of floating macroplastic transport

We quantified floating macroplastic using a camera (GoPro Hero 9, GoPro, San Mateo CA, USA), which was portable, weather resistant, and had a resolution of 1080 × 1920 pixels. The camera had around a 3.5‐hour battery life which we extended using an external battery pack. We also used a 256‐gigabyte secure digital (SD) card, which was the maximum size usable by the camera model. The external battery combined with the large storage card allowed us to record for 10–14 hours. We created a housing for the camera made of wood to attach the camera in an orientation that pointed directly at the river surface and protected the electronics from the weather (Figure S2). To deploy the camera, we attached the system to a vertical railing on the bridge using a bike lock and zip ties. For the one site without a bridge, a metal fence post was placed in the middle of the stream, and the camera stand was attached to the post. The camera was deployed directly above the thalweg of the stream.

### Calibration of the camera‐based data collection method

Previous studies using eyewitnesses or cameras to quantify floating macroplastic in rivers assume an equal distribution of litter across the river width (González‐Fernández et al., 2021). However, our prior observations of macroplastic transport at the study sites suggested aggregation of materials in the thalweg, and therefore assuming even lateral distribution of macroplastics could bias plastic export. Thus, we conducted an in situ calibration to facilitate the extrapolation of measurements collected on camera to the entire stream width.

We completed measurements at four sites in the North Branch Chicago River during spring 2022. First, we set up the camera on the bridge and calibrated the scale of its field of view by directing a researcher to enter the camera frame and place a meter stick on the surface of the water at the start of the recording. Next, we deployed a floating boom downstream of the camera to capture the macroplastic used in the calibration. One researcher moved a minimum of 30 m upstream of the camera, and individually released macroplastic items at random locations across the stream width. We used eight macroplastic item types (Table S2) that represented common macroplastic items in local rivers (McCormick & Hoellein, 2016). We released five replicates of each during the calibration (N = 40 items). The minimum size particles used in calibration were bottle caps around 3 cm in diameter. In the laboratory, we watched the footage to document the number, size, and colors of macroplastic items recorded in the camera view. We assumed the 30 m travel distance between the release point and the camera facilitated the enactment of hydrologic forces which would affect a typical macroplastic item that enters the stream prior to reaching the camera. At the narrowest site, 78% of the stream width was covered by the camera, but 100% of the added items were detected on camera (Figure S3). At the widest site, about 19% of the stream width was covered by the camera view, and 42% of the added items were recorded. This shows that macroplastic in transport was not evenly distributed but was biased towards the thalweg (where the camera was placed). We used linear regression between the proportion of added items that were detected on camera view relative to the proportion of the stream width captured by the camera across four sites was significant (R^2^ = 0.915, p = 0.044) with a line of best fit: ((Items Detected) = 1.048(Stream Width) + 0.2479; Figure S3). Rather than assume an equal distribution of floating macroplastic across stream width, we used this equation to extrapolate from the number of items detected on camera to the stream width for all data collection events. We note the calibration was collected in the North Branch Chicago River watershed but was used to calibrate data collection at all three watersheds. We acknowledge the lack of direct calibration within each watershed but suggest the calibration allows for a conservative estimate of floating macroplastic transport across the river widths.

### Measuring macroplastic during storm events

We used local weather forecasts to predict when a storm would be entering each watershed. We defined a storm as > 10 mm of rain within 12 hours. When possible, we set up the camera and began recording before the rain was forecast to begin. We placed the camera above the thalweg to best capture floating macroplastic. We used flow gauges at each site to monitor the discharge. Each subsequent morning after the camera was set up; the camera SD card and external battery were replaced to start another day's recording. We continued recording the surface of the water until after the river returned to a similar discharge to before the rain started. Measurements could only be taken during the day. Our intention was to capture at least one storm event at each study site (N = 12). However, challenges related to variable weather, site accessibility, drought (i.e., Ipswich River), and equipment operation resulted in 18 events measured at 10 sites (Table 1).

**TABLE 1:** Summary of all storm events.

Storm event	Water‐shed	Water‐shed position	Start date	End date	Days in hydro‐graph	Shape	Peak	Pre‐storm Q (m3/s)	Rising slope (m3/s)/min	Peak Q (m3/s)	Peak Q: pre Q	Falling slope (m3/s)/min
BH2	Chicago	3	22.07.14	22.07.20	7	1 peak	1	0.456	0.0259	7.844	17.205	−0.0164
GM1	Don	1	22.08.04	22.08.06	3	1 peak	1	0.207	0.0071	0.631	3.05	−0.001
GW2	Chicago	2	22.08.28	22.09.07	11	1 peak	1	1.274	0.0039	8.353	6.556	−0.0015
IP062	Ipswich	2	22.10.13	22.10.17	5	1 peak	1	0.046	0.0005	0.883	19.379	−0.0005
SR1	Chicago	1	22.06.08	22.06.10	3	1 peak	1	0.181	0.0017	0.895	4.937	−0.0011
SR2	Chicago	1	22.08.03	22.08.05	3	1 peak	1	0.068	0.0007	0.297	4.375	−0.0005
SR3	Chicago	1	22.09.10	22.09.23	14	1 peak	1	0.063	0.013	7.532	119.283	−0.0009
PR1	Don	3	22.07.17	22.07.20	4	1 peak	1	1.53	0.1138	37.1	24.248	−0.0339
PR2	Don	3	22.11.11			1 peak	1	1.4	0.0444	13.6	9.714	−0.0094
TC2	Don	1	22.09.27	22.09.29	3	1 peak	1	0.344	0.0916	11.339	32.933	−0.0172
SB1	Ipswich	1	22.07.14	22.07.15	1	1 peak	0	‐	‐	‐	‐	‐
BH1	Chicago	3	22.07.04	22.07.08	5	2 peak	1	0.259	0.0565	15.518	59.891	−0.0079
							2	‐	0.0103	12.997	50.164	−0.0062
BH3	Chicago	3	22.08.08	22.08.12	5	2 peak	1	0.796	0.0012	2.554	3.21	−0.0006
							2	‐	0.0013	2.557	3.214	−0.0004
GW1	Chicago	2	22.08.19	22.08.24	6	2 peak	1	0.524	0.0116	3.313	6.324	−0.0033
							2	‐	0.0016	2.973	5.676	−0.0016
TC1	Don	1	22.09.23	22.09.26	4	2 peak	1	0.137	0.0542	9.887	72.198	−0.02
							2	‐	0.1687	11.505	84.01	−0.0194
YM1	Don	2	22.08.20	22.08.24	5	3 peak	1	2.64	0.0243	11.4	4.318	−0.0083
							2	‐	0.0844	52.7	19.962	−0.0535
							3	‐	0.36	39.8	15.076	−0.0303
IP201	Ipswich	3	22.11.11	22.11.12	2	Plateau	1	0.121	0.0002	0.264	2.187	‐
IP061	Ipswich	2	22.07.05	22.07.06	2	Baseflow	0	‐	‐	‐	‐	‐

Watershed position codes: 1 = tributary, 2 = mid‐watershed, and 3 = downstream. Q = discharge, “‐” = no data.

We used a VLC media player (VideoLAN Organization, Paris, France) to analyze a total of 403.5 hours of usable footage in the laboratory. We watched all footage at four to eight times enhanced speed to make processing time effective. While watching the footage, we counted, categorized, and estimated the size of items of macroplastic on the stream surface. For categorization, we used a classification scheme that organizes items material type, and usage category (Figure S4), developed for the larger project of which this study was one part (Haney et al., In press; Schwenk, 2024). We used the meter stick calibration at the start of each recording to measure particle size as described above. Plastic items were counted conservatively. If a particle looked potentially natural, or non‐plastic (e.g., glass or metal), it was not counted. The smallest items recorded were around 1 cm × 1 cm. Small particles like this were able to be counted due to having unnatural colors such as blue. We also recorded the time each item was detected and the total amount of usable footage that day (i.e. the number of hours of footage recorded before nightfall; Table S3). We acknowledge methodological limitations of visual measurements, including detection challenges that occur, variations in the lighting/time of day, transparency of materials, and missing items that are submerged.

We obtained discharge from the stream gauges and divided each storm event into four phases. The time from the start of the recording, until the discharge started to increase, was classified as ‘before’. The time from when the discharge began to increase until it peaked was classified as ‘rising limb’. The time from when the discharge started to drop rapidly to when it started to level off near base level was classified as ‘falling limb’. “After” was classified as the period when discharge was slowly dropping or had fully returned to base level. Determining the transition between ‘falling limb’ and ‘after’ was the most challenging part of the assessment, as the delineation was not always obvious if the falling limb showed a slow recession to baseflow. To accomplish this, we used a log scale plot of the hydrograph and visually determined the point where the slope became linear as the discharge was falling (Dingman, 1994). We also determined the storm flow ratio of the events by dividing the peak discharge by the pre‐storm discharge and defined the rate of change of the storm event as the slope of the rising limb. We noted how many peaks were in each hydrograph and assessed all storm event characteristics (i.e., phase, storm flow ratio, and speed) for each (Table 1).

### Data analysis

We used R [version 2023.09.0 + 463 “Desert Sunflower”] for data analysis (R Development Core Team, 2023). We calculated macroplastic transport rates (No./time) and compared the amount and size of macroplastics documented during each of the storm event phases at each site. First, we adjusted the macroplastic counts for calibration correction using a linear regression analysis (Figure S3), using the *lm*() R function, between the proportion of stream width covered by our camera and the proportion of macroplastics detected. We used Kruskal–Wallis R function (*kruskal.test()*) to compare the macroplastic transport rate (No./30 min) among different phases of each hydrograph. When a significant difference was detected, we used Dunn's multiple comparison tests R function (*dunn. test()*) from [dunn. test] (Dinno, 2024) to determine differences among phases. We repeated this process to compare macroplastic particle size, quantified as area (i.e., length times width) among hydrograph phases. We used 30‐minute intervals to measure transport rate as some rising limbs were relatively brief and the transition from pre‐storm to rising limb to falling limb can occur in a short period of time. Thus, capturing data in 30‐minute segments instead of 1‐hour segments allowed for greater temporal resolution of plastic transport dynamics across the hydrograph.

We next determined the relationship between macroplastic transport rates and storm event size, quantified as both magnitude (i.e., storm flow ratio) and speed (i.e., the slope of the rising limb). First, we calculated the number of macroplastics transported during each storm event. To do so, we used the average macroplastic transport rate (No./time) of the rising limb and multiplied it by the amount of time the rising limb lasted. We repeated this for the falling limb and summed the two values. We used the linear regression R function (*lm)* to quantify the relationship between the total amount of macroplastic transported during each storm event relative to the storm flow ratio and repeated the analysis for storm event speed (log‐transformed). We repeated regressions with each watershed separately, and with watersheds combined.

Last, we used the equation from the positive relationship between storm magnitude and total macroplastic export to complete a discharge‐weighted calculation of macroplastic export for the North Branch Chicago River at Bunker Hill, the most downstream site, in summer 2022. We chose the Chicago River because there was no macroplastic export from the Ipswich River, and the Don River calculations were completed for a separate study (Haney et al. In Press). We obtained the hydrograph with discharge measured at 15‐minute intervals from June 1 to August 31, 2022 via the United States Geologic Survey (Gage number 05536000). We used the same methods as described above to distinguish baseflow from rising and falling limbs for each of the storm peaks during that time period. We calculated the total amount of time at baseflow. We used the mean macroplastic transport measured during the pre‐ and post‐storm periods to calculate the mean rate of macroplastic transport (No./hour) at baseflow. We then multiplied the mean transport rate (No./hour) by the hours at which the stream was at baseflow, which showed how many floating macroplastic items were exported from the river during baseflow. Next, we calculated the storm flow ratio for each storm event that occurred during the summer. We used the predictive relationship between storm flow ratio and macroplastic transport (generated via the analysis described above) to calculate the number of macroplastics that were exported from each storm event and summed across all storm events. All data, as well as the R code for analysis and figures, is publicly available (Hoellein & Schwenk, 2024).

## RESULTS

### Macroplastic transport across storm event hydrograph phases

**FIGURE 2:**
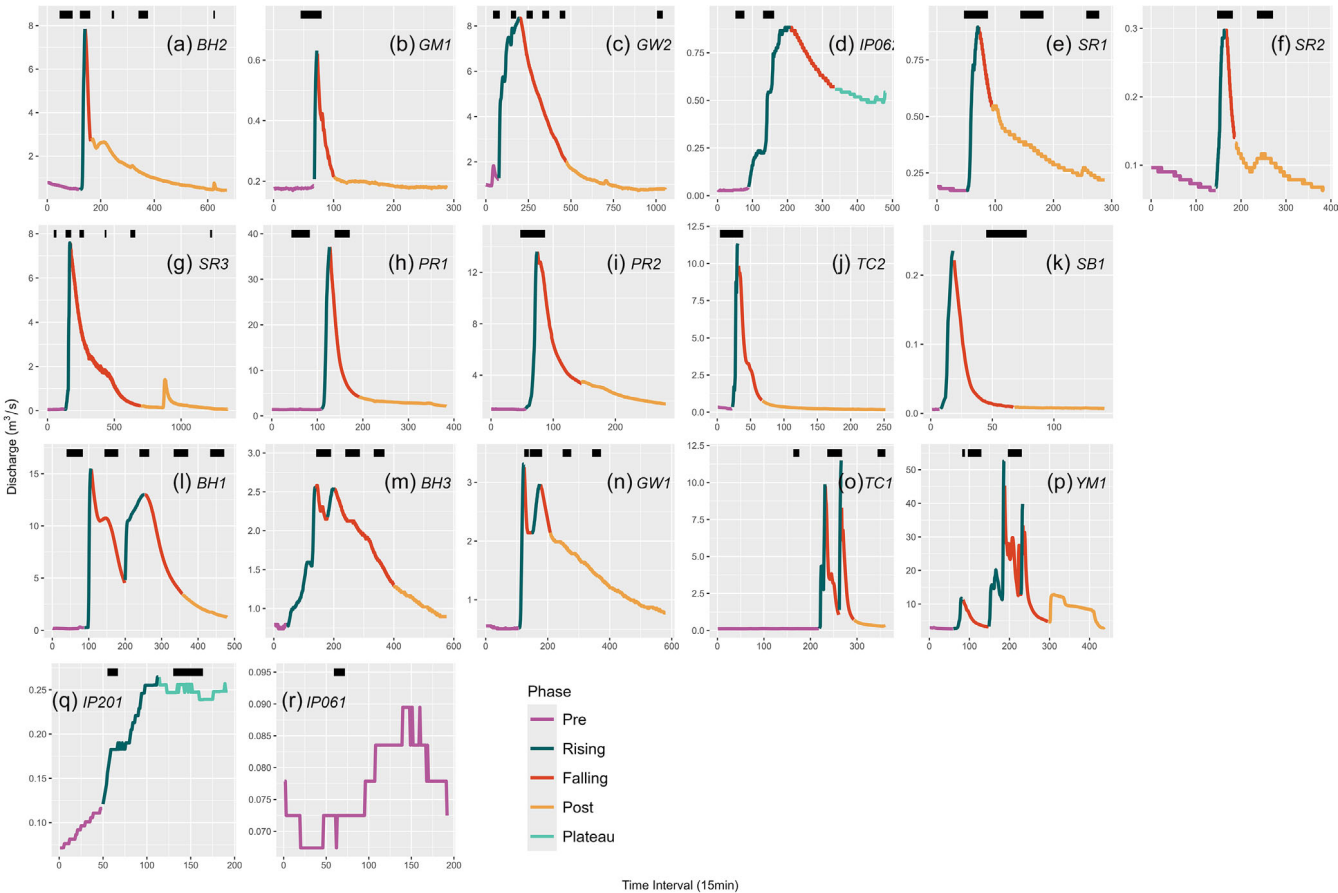
Hydrographs for 18 storm events used in this analysis. Time was recorded 15‐minute increments. Events with one peak are in the first 2 rows (A‐K), events with 2 peaks are in the (plots L‐P), those that did not fit the previous 2 categories are in the fourth row (plots Q‐R). Storm phase is depicted by color. Black bars represent video data collection.

The storm events successfully captured on camera (N = 18) were highly variable in magnitude, duration, and timing (Figure 2, Table 1, and Table S3). The most common hydrograph showed a single peak (N = 11; Figure 2A‐K), some showed multiple peaks (N = 5; Figure 2L‐P), and two hydrographs did not have a clear peak (N = 2 Figure 2Q‐R). The pattern in the latter two examples was due to a drought in the Ipswich River watershed in 2022. The amount of camera footage captured across the different phases of the hydrographs was also variable due to the unpredictability of weather events, battery life, researcher availability, and timing of the hydrograph at night vs day (Figure 2).

**FIGURE 3:**
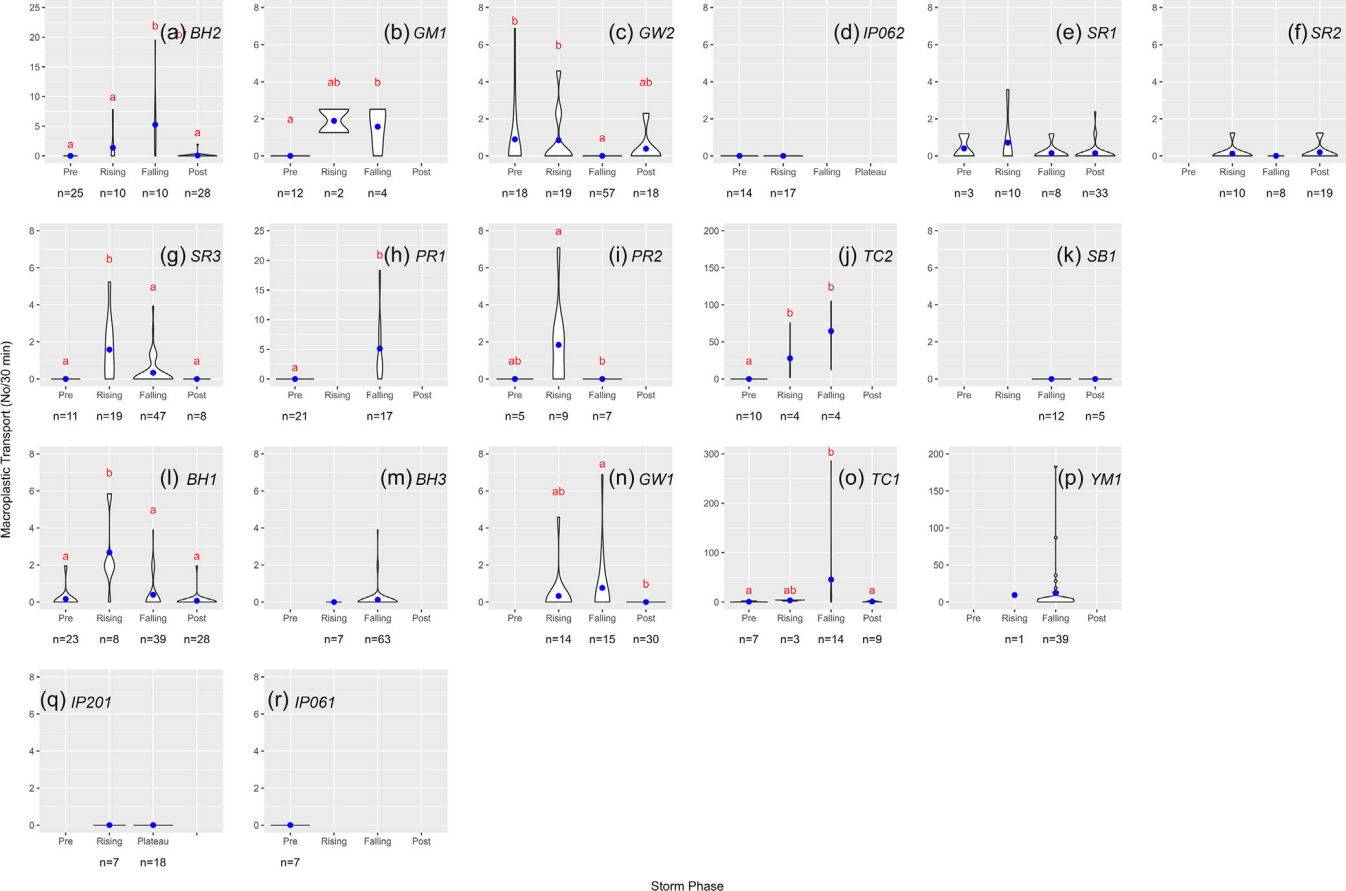
Macroplastic transport rates by storm phase (points = mean). The total number of 30‐minute segments of video footage for each phase is shown below the plots. Small letters above show differences among phases (Dunn's multiple comparison test).

Macroplastic transport rates (No items/30 min) varied across storm event phases (pre‐storm event, rising limb, falling limb, and post‐storm event). We observed 936 macroplastics in total. Transport rates data ranged from 0 to 285.6 items/30 min, with the Don River events having higher transport rates than the other watersheds. During nine of the 18 storms, macroplastic transport rate was significantly higher during the rising and/or falling limb relative to pre‐ and post‐storm (p < 0.01; Table 2). Of those nine storms, three showed significantly more macroplastic transport during the rising limb than other phases (Figure 3G, J, and L), three showed higher transport during the falling limb than other phases (Figure 3A, H, and O), and three showed higher transport in both falling and rising limbs relative to other phases (Figure 3B, J, and N; Table 2). The remaining nine storm events did not show any difference in macroplastic transport during storms compared to baseflow. There was also no difference in macroplastic size relative to the storm phase at 16 of 18 storms (Table S4). The two storms that showed differences showed variable patterns (Figure S5).

**TABLE 2:** Kruskal–Wallis test results for comparing macroplastic transport rates (no/30 min) among storm phases.

Site/storm	Water‐shed	Water‐shed position	Transport rate (no/30 min)		
			Test‐stat	p‐value	Df
BH2	Chicago	3	33.66	<0.01	3
GM1	Don	1	13.02	<0.01	2
GW2	Chicago	2	14.81	<0.01	3
IP062	Ipswich	2	N/A	N/A	1
SR1	Chicago	1	2.06	0.56	3
SR2	Chicago	1	1.43	0.49	2
SR3	Chicago	1	23.52	<0.01	3
PR1	Don	3	25.1	<0.01	1
PR2	Don	3	8.24	<0.01	2
TC2	Don	1	15.75	<0.01	2
SB1	Ipswich	1	N/A	N/A	1
BH1	Chicago	3	33.5	<0.01	3
BH3	Chicago	3	0.34	0.06	1
GW1	Chicago	2	6.15	<0.01	2
TC1	Don	1	19.24	<0.01	3
YM1	Don	2	1.21	0.27	1
IP201	Ipswich	3	N/A	N/A	1
IP061	Ipswich	2	N/A	N/A	0

Events with one clear peak are listed first, followed by events with multiple peaks, then other shapes. Watershed position codes: 1 = tributary, 2 = mid‐watershed, and 3 = downstream. P‐values <0.05 are in bold. N/A means no plastics were observed during the event.

We compared the categories of floating macroplastics transported relative to the storm event phase (Figure S6). The most common items were non‐polystyrene plastics (e.g. bottles and food wrappers), which made up 57.6% of the items, and expanded polystyrene (EPS) plastics, which made up 36.8% of the items. Hygiene products and other miscellaneous forms of plastic represented 1.5% and 2.5% of all items encountered. There was no pattern between the storm event phase and the categories of macroplastic noted (Figure S6).

### Macroplastic transport relative to discharge

**FIGURE 4:**
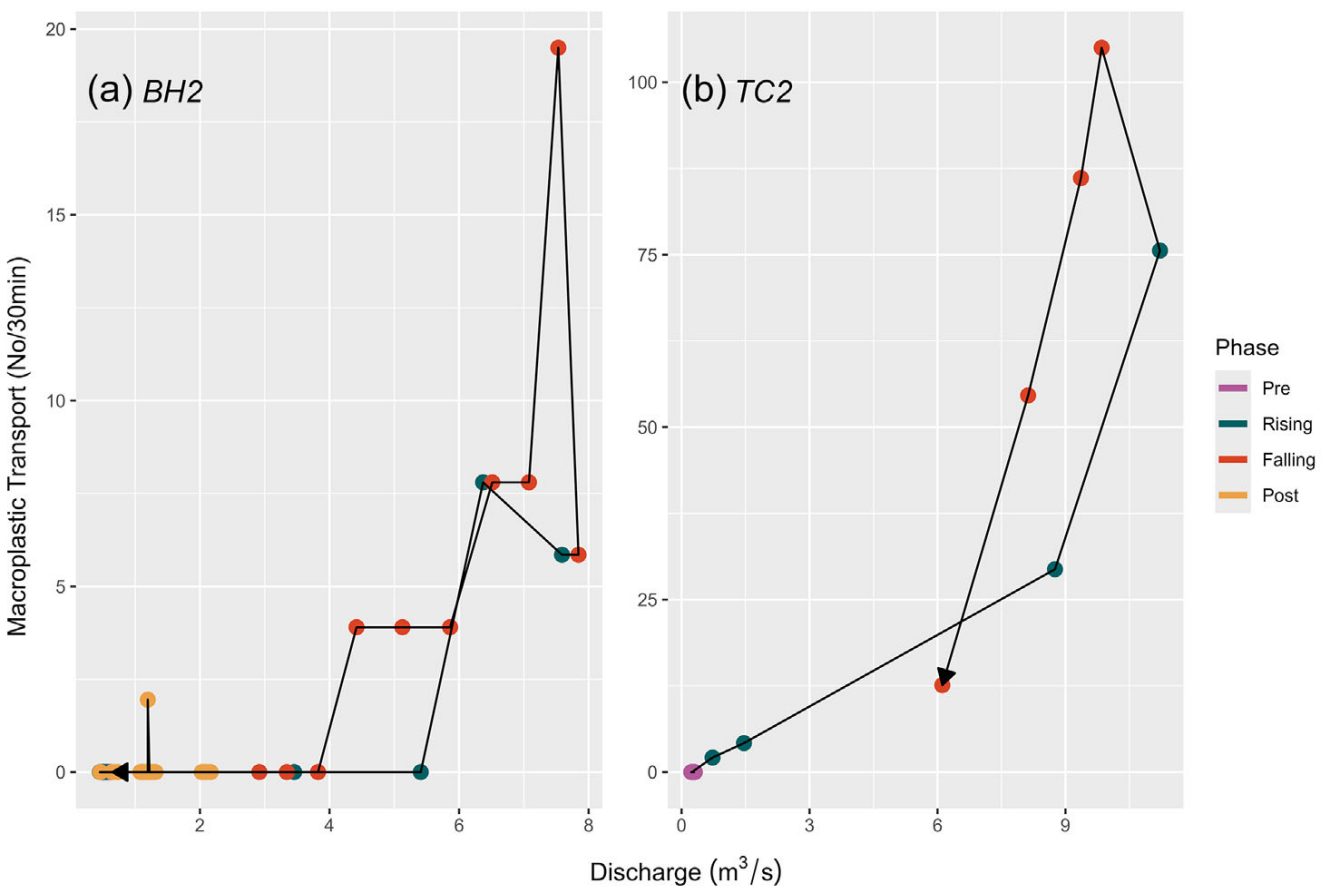
Macroplastic transport rate by stream discharge for two events which suggested hysteresis. The color of the points represents the storm phase. The line connects the points in order of occurrence.

Comparing macroplastic transport to discharge showed that in 16 of the storm events, macroplastic transport rates were unrelated to discharge and storm event phases, with no evidence for hysteresis (Figure S7). However, two of the events showed patterns suggesting potential hysteresis (Figure 4A and B). The events had elevated plastics in the falling limb of the storm in relation to the rising limb when at similar discharges. This could indicate counterclockwise hysteresis. We repeated the analyses for particle size relative to discharge, which showed no relationships for any of the storm events (Figure S8).

**FIGURE 5:**
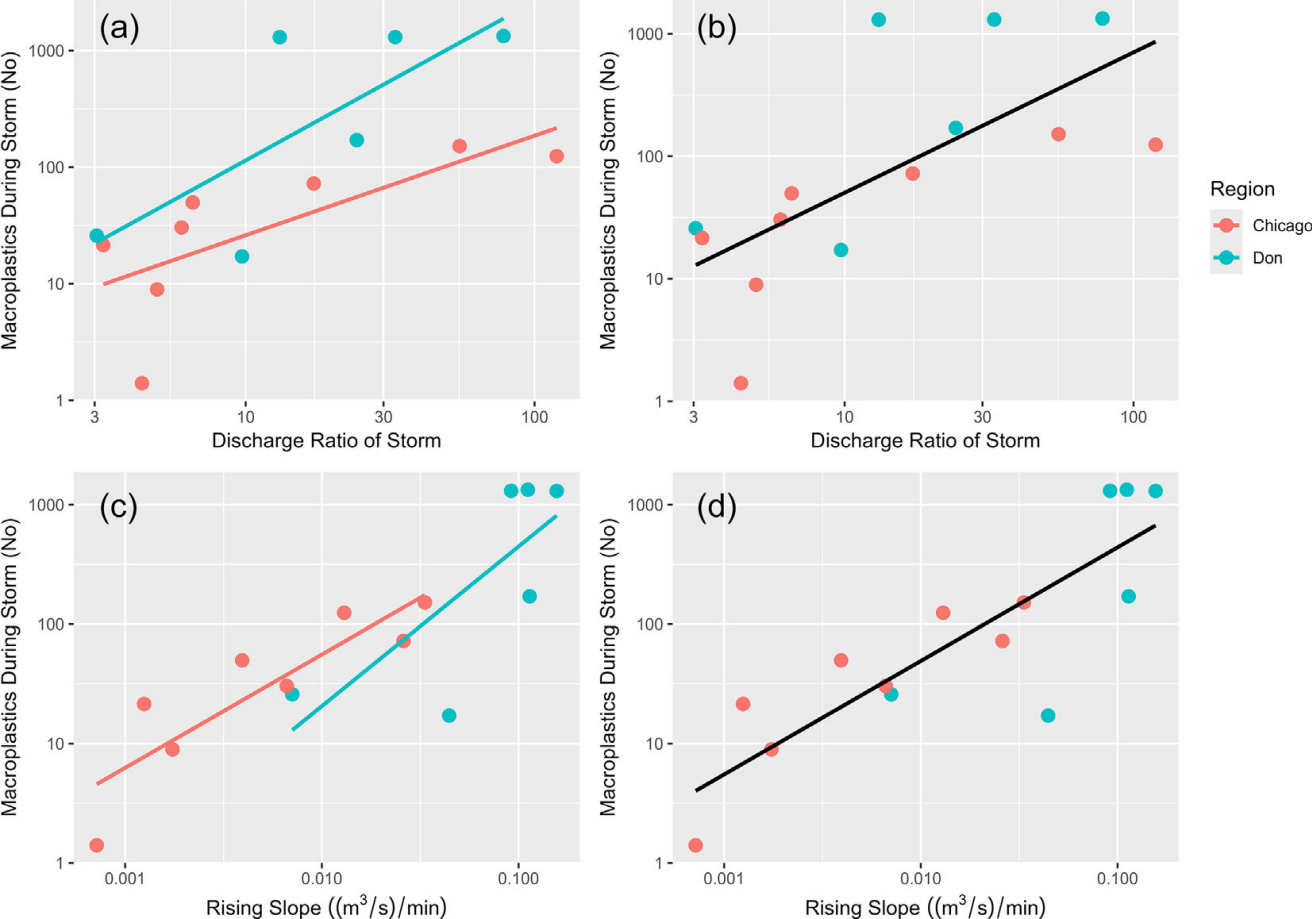
Storm magnitude (storm flow ratio) relative to total macroplastics transported A) in Chicago and Don Rivers individually, and B) all data combined. Storm speed (rising slope) in C) Chicago and Don Rivers individually, and D) all data combined.

We next determined how the magnitude and speed of storm events related to floating macroplastic transport (Figure 5). The storm flow ratio showed a positive relationship to number of macroplastics transported when considering the North Branch Chicago River alone (R^2^ = 0.531; p = 0.040; Figure 5A), the Don River alone (R^2^ = 0.558; p = 0.088; Figure 5A) and the combined Don and Chicago River storm events (R^2^ = 0.474; p = 0.006; Figure 5B). Maximum macroplastic export was about 10 times higher from the Don River relative to Chicago River sites (Figure 5A) and no macroplastics were observed in transport in the Ipswich River. The rising slope also showed a positive relationship with the number of macroplastics moved during storm events in the North Branch Chicago River alone (R^2^ = 0.730; p = 0.004; Figure 5C), the Don River alone (R^2^ = 0.564; p = 0.085; Figure 5), and the combined Don and Chicago River data (R^2^ = 0.729; p < 0.001; Figure 5D). Collectively, these data demonstrated macroplastic transport rates are predicted in part by hydrologic metrics of storms when compared across sites and events.

Last, we used the positive relationship between macroplastic and storm flow ratio for the Chicago River (Figure 5A) to calculate the total amount of macroplastics exported from the North Branch Chicago River at Bunker Hill from June 1 to August 31, 2022 (Figure S9). We calculated that 2721 floating macroplastic items were exported during that time (Table S5, Table S6). Of those items, 80.6% were exported during storm events, while 19.4% were exported during baseflow. However, storm events represented only 25.2% of the total time during that interval, while base flow was 74.8% of the time.

## DISCUSSION

Our dataset of macroplastic transport measurements across multiple sites and storm events allowed for inferences on the relationship between hydrograph phase and storm event magnitude with plastic transport dynamics. Results showed no clear evidence of a first‐flush pattern or hysteresis, although the data suggested greater macroplastic transport in more urban sites which could influence the capacity to detect among‐phase patterns in transport rates (Roebroek et al., 2024). Across all storm events, a positive correlation between both storm event magnitude and speed with macroplastic transport can facilitate discharge‐weighted calculations of plastic transport at the watershed scale over longer time scales.

### Macroplastic transport: storm event phases and hysteresis

We expected macroplastic transport rates during storm events (i.e., rising and falling limbs of hydrograph) to be significantly higher than baseflow conditions, however, patterns varied among sites and events. Data from half of the storm events (N = 9 of 18 total) showed support for our prediction while the other nine events showed no differences among phases. One key difference between these two groups was storm event magnitude. The storm events that supported our predictions had a significantly higher mean (±standard deviation) storm flow ratio (peak/pre‐storm event discharge) of 39.6 (±40.1), relative to the storms storm events that did not show this relationship, which had a mean (±standard deviation) storm flow ratio of 8.6 (±7.6) (t‐test, t = 2.00, p = 0.03). The patterns suggest larger storm events could increase macroplastic transport rates more than smaller storm events, likely through enhanced input and mobilization.

Macroplastic transport rates did not show hysteresis, which we had expected would offer insight into the timing of macroplastic input to streams and how macroplastics already in the stream channel are remobilized during storms. A clockwise hysteresis would suggest pollutants come off the landscape and enter streams at the start of a storm (Carey et al., 2014; Deletic, 1998; Lee et al., 2002). We did not find that pattern, which may indicate that macroplastics that were transported during storms are already in the stream or immediate riparian zone. In addition, two storm events demonstrated counterclockwise patterns, which may suggest that macroplastics transported during the storm events were not added to the stream during the first flush of stormwater. Instead, those items could have been macroplastics that were already entrained in the stream channel and were lifted into the water column at later points during the storm. This could include macroplastics that were mobilized as the riparian zone became flooded, or plastics that hung in vegetation above the stream channel at low flow and entered the flood waters later in the storm. Overall, the hysteresis dynamics of macroplastic transport documented here allow for only limited inferences about its source and transport dynamics within river channels, and more data are needed to quantify if hysteresis is a feature of macroplastic movement in urban streams.

Previous research on hysteresis in the transport of pollutants in rivers has typically focused on the movement of dissolved chemicals and suspended solids in flowing waters, rather than large floating particulates such as macroplastic. For example, Deletic (1998) collected storm runoff from asphalt streets during the growing season in two small urban catchments in Yugoslavia and Sweden. The authors found the first flush pattern only occurred for suspended solids, and not for solutes (e.g., conductivity or pH). Lee et al. (2002) measured stormwater runoff from 38 storms across 13 urban watersheds and found that the first flush pattern was strongest for suspended solids from residential areas and for measurements collected in smaller watersheds. Finally Cowger et al. (2022) measured macroplastic transport at rising and falling limbs of two storms for a stream in southern California, USA, and their data showed potential evidence for counterclockwise hysteresis during one storm, and clockwise hysteresis during the other. These patterns have a useful bearing on expectations for hysteresis patterns of macroplastic transport in urban streams. If macroplastic transport follows a similar pattern as previous studies, then it is possible that only smaller particles may show a first flush effect, and the pattern may be strongest in smaller watersheds. Although, patterns may vary depending on hydrologic context and the material composition, even for storms that occur at the same site (Cowger et al., 2022). More research is needed to offer quantitative assessments of factors that drive macroplastic transport in urban streams. Specifically, future studies would benefit from examining macroplastic transport hysteresis across gradients of watershed characteristics (e.g., land cover, watershed size), storm features (e.g., magnitude, duration, and time since preceding storms), and physical aspects of the particles in transport (e.g., size, density, and shape).

We found that both stream discharge and urban land use were related to patterns of plastic transport, consistent with previous research which compared macroplastic transport rates at high and low discharge and spanning various time scales. For example, van Emmerik et al. (2019) used data collected by eyewitnesses stationed on bridges of the Seine River, France to quantify macroplastic transport. The authors noted macroplastic transport was greater during high discharge (301 items/30 min) relative to low discharge (80 items/30 min), and rates were higher downstream of Paris relative to more rural upstream sites. Rates measured in that study were higher than this study, although in the same order of magnitude reported here (0–286 items/30 min). In addition, our least urban site had the lowest macroplastic transport rates (Ipswich River), and the most urban site had the greatest transport rates (Figure 5). Treilles et al. (2022) also examined microplastic flux in relation to flooding in the Seine River, estimating that flooding from January to February 2018 contributed 40% of the annual microplastic flux but was only 14.5% of the year. Other analyses have compared the macroplastic at high and low flows relative to individual storm events. van Emmerik et al. (2023) measured the transport of floating macroplastic during a single major flood in the Meuse (Netherlands) and found that it was 141 times higher than pre‐discharge transport. Collectively, the data suggest that increased discharge and urbanization, which likely enhance total macroplastic availability, each contribute to elevated transport rates in rivers. In addition, the magnitude of macroplastic transport rates is similar across studies, despite variations in detection methods and hydrologic context.

### Macroplastic size and storm event phase

Contrary to our expectations that macroplastic particle size would be larger at high discharge, hydrograph phase was unrelated to macroplastic size. Only 2 of 18 storm events showed a difference in macroplastic size among hydrograph phases, with different patterns for each. We attributed this lack of variation in size to differences in macroplastic density by polymer and a potential methodological artifact. Our prediction that larger particles would be observed at high discharge assumed a relationship between particle size and capacity for resuspension that does not uniformly apply to different types of plastic which have variable densities. For example, polyvinyl chloride (PVC) has a density of about 1.4 g/cm^3^ and low‐density polyethylene has a density of about 0.9 g/cm^3^ (Borges‐Ramírez et al., 2020). A small piece of PVC may require a large amount of stream velocity to move, while large pieces of polyethylene could be mobilized at lower water speeds. This discrepancy could obscure any relationship between the storm event phase and macroplastic size. In addition, our measurement of macroplastic size was based on a calibration at baseflow. The surface of the water was farthest from the camera when the calibration took place, and closer as the stream height increased. The reduced distance to the camera could overestimate particle size during high discharge.

Previous research focusing on the size of floating macroplastics transported in rivers is relatively limited. Pogojeva et al. (2023) used eyewitnesses on bridges to monitor three tributaries of the Black Sea during baseflow and found most items were <10–20 cm, with plastic as the most common material (74% of items). The authors concluded that most of the macroplastics transported at baseflow in their sites were small and lightweight. In our dataset, we found a median (+/− std dev) particle size was 64 (+/− 470) cm^2^. Thus, 50% of the items we observed were <64 cm^2^ (i.e., approximately 8 × 8 cm). The high standard deviation was caused by the large items which were relatively uncommon. However, our median size is consistent with the results of Pogojeva et al. (2023), where most items were < 20 cm. We also note that additional data collection methods are likely needed to accurately quantify small macroplastic particles (i.e., 0.5–5 cm), which present a detection challenge for eyewitness and camera‐based methods. Future studies will benefit from coordinated methods such as the combined use of visual detection with sample collection via nets or pumps. These methodological advancements will better capture the full size range of macroplastic transported during storm events (Vriend et al., 2020), and thereby generate stronger inferences of how macroplastic particle size may relate to change in discharge.

### Composition of macroplastic varies among sites and hydrograph phases

The categories of macroplastics observed in transport showed no apparent pattern among sites or hydrograph phase and no clear signature of combined sewer influences. The items most encountered were fragments and packaging, although the composition of individual item categories varied across all storm phases of each hydrograph (Figure S6). We attribute this variation to the difference in number of items and the lack of identification by polymer type. For hydrograph phases with a lower number of items detected, the identity of the individual items has a strong impact on the relative abundance of macroplastics by category. In addition, we conducted a visual identification and did not collect particles for polymer identification. It is possible that polymer type (e.g., material density) may be more strongly related to the hydrograph phase than the particle category. Finally, hygiene products represented 1.5% of all items observed by our cameras across all sites, which does not support our original hypothesis that hygiene products would be higher due to CSO (combined sewer overflow) in the Chicago and Don Rivers. Linking CSO events to our data collection sites was challenging, however, as municipalities report that an event occurred in a watershed without details on the specific location or pipe outfall. Sewage‐related macroplastics are influenced by storms in urban streams elsewhere. For example, Williams and Simmons (1999) measured anthropogenic litter at 50 stream sites in the UK and found that feminine hygiene products were 22% of all items recorded. The abundance of hygiene products was related to proximity to drainage pipes, suggesting they came from combined sewers. Given the individual events captured and the assemblage of litter in this study, however, we concluded that storm events generally remobilized particles already present in the stream and riparian zone, rather than changing the composition of macroplastics in transit.

Past studies have found similar patterns in the composition of riverine macroplastics, although we are not aware of any analyses comparing assemblage across hydrograph phases. Vriend et al. (2020) combined visual observation of macroplastic transport rates from a bridge with passive sampling using shoreline litter traps to characterize macroplastic in the Rhine River in Europe. The authors reported polyolefin items were the most common type of litter, which includes polyethylene and polypropylene. That pattern is consistent with the composition of plastic categories recorded in our dataset. Pogojeva et al. (2023) found that the most abundant material types in three tributary rivers of the Black Sea were artificial polymers and paper/cardboard. The authors noted the most common item types were similar to categories noted in our sites, including plastic fragments, bottles, packaging, and bags.

### Storm magnitude and speed were related to macroplastic transport

Storm magnitude (i.e., storm flow ratio) and speed (i.e., slope of the rising limb) were each related to total macroplastic transport, and both metrics of storm strength offer complementary insights into macroplastic transport. The positive relationship between storm flow ratio and macroplastic transport (Figure 5A, B), suggests that a critical driving factor of macroplastic movement is the peak discharge attained. We infer from this evidence that large storm peaks could mobilize more macroplastic, and could do so through several potential mechanisms, including flooding a greater surface area of the riparian zone, moving litter into the stream channel, and generating the hydrological force to move it downstream. The relationship with the slope of the rising limb suggests speed of storms also affected macroplastic transport. A storm event that takes longer to reach a peak discharge might move less macroplastic if it allowed particles to be entrained when water is rising, rather than rapidly moving macroplastics downstream in a flash flood. In this dataset, we cannot separate the role of each factor, although additional research across a range of storm conditions will allow for a greater understanding of the contribution of storm magnitude and storm speed on macroplastic transport.

Finally, the predictive equations we generated between macroplastic export and both storm flow ratio and rising limb slope can be used to estimate plastic export over longer time scales. Our result for the North Branch Chicago River, where 81% of macroplastics were exported during storms, which occurred only 24% of the time, identifies storm flows as the key moments of trash export. This approach improves on previous estimates because it is a discharge‐weighted calculation, and can be used to generate key parameters to inform a plastic budget at the watershed scale (Hoellein & Rochman, 2021). A discharge‐weighted approach is needed to reduce the large uncertainty that currently impedes watershed‐scale models of plastic export from rivers (Roebroek et al., 2022). In addition, the results suggest that the deployment of trash interception devices in rivers will be most successful when in operation at high flow.

### Methodological considerations for future studies

The visual detection method used here and in previous research has provided valuable data to measure the transport of macroplastic and other debris in rivers but can benefit from potential improvements in future analyses. More robust data could be collected with added technology such as night vision capacity for cameras or eyewitnesses. The simultaneous deployment of nets or passive samplers just downstream of the camera could collect floating and submerged debris while recordings are ongoing, allowing researchers to compare transport rates to those detected at the surface (Cowger et al., 2022; Vriend et al., 2020). Additional checks on the video recordings could also provide more robust data, such as multiple viewers of the same footage and the use of artificial intelligence to conduct measurements (Gnann et al., 2022). To improve particle size measurements, we suggest future studies can quantify the distance from the camera to the stream surface at variable stream heights. Finally, more robust particle size metrics will benefit hysteresis analysis. In particular, our analysis did not discriminate across subclasses of macroplastic sizes or material types in the plots of discharge vs transport (Figure S5, Figure S8). We suggest studies that examine macroplastic transport in smaller size classes (e.g., 5–50 mm) separate from larger classes, or those that can collect macroplastic in transport to examine polymer composition, may uncover different patterns of hysteresis. Overall, we expect instrumentation and analytical advancements such as these will refine empirical data collection on riverine transport of macroplastics and other anthropogenic debris.

### Conclusions

Measuring stream macroplastic transport during 18 storm events, across three watersheds, and covering a gradient of storm sizes revealed quantitative relationships for macroplastic movement during storm events. Our analysis of macroplastic transport across different phases of storm events, instead of just before and after, did not offer evidence for the hysteresis of macroplastic transport. The data suggested macroplastic transport during storms may not follow the first flush phenomenon shown for some other pollutants. However, we acknowledge the patterns in the dataset were influenced by variations in storm size, macroplastic size, and macroplastic composition, which could be addressed in future studies. Despite the variability, our data generated significant predictive equations between storm size and total macroplastic transport, which demonstrated the critical role of storms on macroplastic export when extrapolated longer time scales (e.g., one summer). Future research can build on this dataset to generate discharge‐weighted estimates of macroplastic export from these watersheds and those with similar hydrologic characteristics. Our results suggest that macroplastic in streams is mobile during storms, so attempts to reduce macroplastic pollution locally could help reduce pollution downstream. In addition, focusing on the role of storms, including different hydrograph phases, as key moments for macroplastic input and transport can help generate prevention, collection, and interception strategies to reduce macroplastic pollution.

## AUTHOR CONTRIBUTIONS

**Bailey A. Schwenk:** Investigation; writing – original draft; conceptualization; methodology; validation; visualization; writing – review and editing; formal analysis; data curation. **Elizabeth M. Kazmierczak:** Investigation; methodology; visualization; writing – review and editing. **Fritz Petersen:** Investigation; methodology; visualization. **Jacob Haney:** Investigation; methodology; visualization; formal analysis. **Xia Zhu:** Investigation; writing – review and editing. **Shan Zuidema:** Investigation; writing – review and editing; methodology; visualization. **Emily K. Lever:** Investigation; writing – review and editing; methodology; visualization; data curation. **Richard B. Lammers:** Funding acquisition; writing – review and editing. **Wilfred M. Wollheim:** Investigation; conceptualization; funding acquisition; writing – review and editing; methodology; visualization; formal analysis; project administration; resources. **Chelsea M. Rochman:** Conceptualization; investigation; funding acquisition; writing – review and editing; methodology; validation; formal analysis; supervision; project administration; resources. **Timothy J. Hoellein:** Conceptualization; investigation; funding acquisition; writing – original draft; methodology; validation; visualization; writing – review and editing; formal analysis; project administration; data curation; supervision; resources.

## CONFLICT OF INTEREST STATEMENT

The authors declare no conflicts of interest.

## Supporting information


**Table S1.** Watershed characteristics size, stream width, and land use. Width = average (std. dev) of 9 measurements at base flow.
**Table S2.** List of item types in calibration item set.
**Table S3.** Summary of video recorded for all floods. Events with one clear peak are listed first, followed by events with multiple peaks, then other shapes.
**Table S4.** Kruskal Wallis test results for comparing macroplastic macroplastic size (m^2^) among storm phases. Events with one clear peak are listed first, followed by events with multiple peaks, then other shapes. Watershed position: 1 = tributary, 2 = mid‐watershed, 3 = downstream. P‐values <0.05 are in bold. N/A means all observed plastics were in the same phase of the hydrograph. A “‐“means there were no observed plastics.
**Table S5.** Total time spent, and items transported by both storms and baseflow conditions from June 1 to August 31, 2022, at the Bunker Hill site in the North Branch Chicago River. Mean items per hour during pre‐ and post‐storm hydrographs were used to estimate plastic transport during baseflow. Discharge ratio and duration of storms were used to estimate the plastic transport of individual storms, which were then combined for the entire summer.
**Table S6.** Data for all flood events between June 1 and August 31, 2022, at the Bunker Hill site in the North Branch Chicago River. Peak and Pre‐flood discharge (Q) were used to calculate each flood's discharge ratio. The discharge ratio was used to estimate macroplastic transport during each flood. We used the line of best fit from our log‐scaled relationship between macroplastic transport and discharge ratio of floods to calculate these values (y = 0.854x + 1.293; y is the log‐scaled number of macroplastic; x is the discharge ratio of the flood).
**Figure S1.** Three study watersheds: North Branch Chicago River (A), Don River (B), and Ipswich River (C). Colors represent different forms of land use for each watershed. Study sites are shown as red circles.
**Figure S2.** The camera mount attached to a pedestrian bridge. The camera was attached under the bottom portion of the mount and recorded the surface of the stream.
**Figure S3.** Macroplastic calibration analysis for video camera particle detection, completed at four sites in the North Branch Chicago River. R^2^ = 0.915, p = 0.044, line of best fit: y = 1.048x + 0.2479.
**Figure S4.** Datasheet used to categorize all collected anthropogenic litter (AL). The first column is the function of the collected item, the second column is the general material type of the AL, and columns 3–6 are the habitats the AL was found in.
**Figure S5.** Violin plots showing macroplastic size by storm phase (points = mean). The total number of items reported for each phase is shown below the plots. Small letters represent differences (Dunn's multiple comparison test) after a significant Kruskal‐Wallis test among phases.
**Figure S6.** The relative abundance of macroplastic categories detected by storm phase. Shades of red represent non‐polystyrene plastics, shades of green represent expanded polystyrene plastics, shades of blue represent hygiene products, and the other colors represent miscellaneous plastics.
**Figure S7.** Macroplastic transport rate by stream discharge, where the colors show the storm phase. Jitter was added to the plots for better visualization.
**Figure S8.** Size of macroplastics by discharge, where point color represents storm phase.
**Figure S9.** Hydrograph for June 1st‐ August 31st, 2022, at Bunker Hill. Date as “month/day” is shown on the x‐axis.

## Data Availability

The data that support the findings of this study are openly available in Mendeley Data at https://data.mendeley.com/datasets/55dnc6c2hm/1, reference number 10.17632/55dnc6c2hm.1.
