# Supplementary material for: Storm events influence the transport of macroplastics in urban streams
Source: Water Environ Res. 2025 Jun 1;97(6):e70083. doi: 10.1002/wer.70083 (PMC12127040; doi:10.1002/wer.70083)
Supplement: Supplementary file 1 — Table S1. Watershed characteristics size, stream width, and land use. Width = average (std. dev) of 9 measurements at base flow. Table S2. List of item types in calibration item set. Table S3. Summary of video recorded for all floods. Events with one clear peak are listed first, followed by events with multiple peaks, then other shapes. Table S4. Kruskal Wallis test results for comparing macroplastic macroplastic size (m2) among storm phases. Events with one clear peak are listed first, followed by events with multiple peaks, then other shapes. Watershed position: 1 = tributary, 2 = mid‐watershed, 3 = downstream. P‐values <0.05 are in bold. N/A means all observed plastics were in the same phase of the hydrograph. A “‐“means there were no observed plastics. Table S5. Total time spent, and items transported by both storms and baseflow conditions from June 1 to August 31, 2022, at the Bunker Hill site in the North Branch Chicago River. Mean items per hour during pre‐ and post‐storm hydrographs were used to estimate plastic transport during baseflow. Discharge ratio and duration of storms were used to estimate the plastic transport of individual storms, which were then combined for the entire summer. Table S6. Data for all flood events between June 1 and August 31, 2022, at the Bunker Hill site in the North Branch Chicago River. Peak and Pre‐flood discharge (Q) were used to calculate each flood's discharge ratio. The discharge ratio was used to estimate macroplastic transport during each flood. We used the line of best fit from our log‐scaled relationship between macroplastic transport and discharge ratio of floods to calculate these values (y = 0.854x + 1.293; y is the log‐scaled number of macroplastic; x is the discharge ratio of the flood). Figure S1. Three study watersheds: North Branch Chicago River (A), Don River (B), and Ipswich River (C). Colors represent different forms of land use for each watershed. Study sites are shown as red circles. Figure S2. The camera [file WER-97-e70083-s001.docx]

Supplemental Materials: Storms influence the transport of macroplastics in urban streams

Bailey A. Schwenk^1,5^, Elizabeth M. Kazmierczak^1,6^, Fritz Petersen^1,7^, Jacob Haney^2,9^, Xia Zhu^2,10^ Shan Zuidema^3,11^, Emily K. Lever^3,13^, Richard B. Lammers^4,12^ Wilfred M. Wollheim^3,14^, Chelsea M Rochman^2,8^, Timothy J. Hoellein^1,15^

^1^Department of Biology, Loyola University Chicago, 1032 West Sheridan Road, Chicago, Illinois 60660, United States.

^2^Department of Ecology and Evolutionary Biology, University of Toronto, 3359 Mississauga Road, Mississauga Ontario L5L 1C6, Canada

^3^Earth Systems Research Center, University of New Hampshire, 8 College Rd, Durham, NH 03824, USA

^4^College of Life Sciences and Agriculture, University of New Hampshire, 105 Main St, Durham, New Hampshire 03824, United States

email: [^5^bschwenk@luc.edu](mailto:bschwenk@luc.edu), ^6^[ekaz0103@gmail.com](mailto:ekaz0103@gmail.com), ^7^[fxp120@case.edu](mailto:fxp120@case.edu), ^8^[chelsea.rochman@utoronto.ca](mailto:chelsea.rochman@utoronto.ca), ^9^[jacob.haney@utoronto.ca](mailto:jacob.haney@utoronto.ca), ^10^[alicexia.zhu@mail.utoronto.ca](mailto:alicexia.zhu@mail.utoronto.ca), ^11^[Shan.Zuidema@unh.edu](mailto:Shan.Zuidema@unh.edu), ^12^[Richard.Lammers@unh.edu](mailto:Richard.Lammers@unh.edu), ^13^[Emily.Lever@unh.edu](mailto:Emily.Lever@unh.edu), ^14^[Wil.Wollheim@unh.edu](mailto:Wil.Wollheim@unh.edu), ^15^[thoellein@luc.edu](mailto:thoellein@luc.edu)

Table S1. Watershed characteristics size, stream width, and land use. Width = average (std. dev) of 9 measurements at base flow.

| Water-shed | Site | Water-shed position | Water-shed Area (km^2^) | Width (m) | Pop. Density (No/km^2^) | Imperv-ious surface (%) | Urban (%) | Forest (%) | Grass (%) | Wetland (%) | Crop (%) | Barren (%) | Water (%) |
| --- | --- | --- | --- | --- | --- | --- | --- | --- | --- | --- | --- | --- | --- |
| Chicago | Bunker Hill | 3 | 271 | 15.0 (1.1) | 1049 | 67.4 | 85.9 | 5.8 | 0.8 | 5.6 | 1.0 | 0.1 | 0.9 |
| Chicago | Glenview Woods | 2 | 157 | 10.9 (0.6) | 728 | 56.5 | 79.9 | 8.3 | 1.1 | 8.1 | 1.2 | 0.1 | 1.4 |
| Chicago | Skokie River | 1 | 26.2 | 5.1 (0.9) | 750 | 58.5 | 81.0 | 7.2 | 0.5 | 9.3 | 1.7 | 0.1 | 0.2 |
| Chicago | Middle Fork | 1 | 32.3 | 6.3 (1.0) | 443 | 48.3 | 64.4 | 12.5 | 2.5 | 14.3 | 4.6 | 0.1 | 1.7 |
| Don | Pottery Road | 3 | 318 | 19.1 (1.8) | 3870 | 79.7 | 80.7 | 9.6 | 2.8 | 0.4 | 4.6 | 1.7 | 0.2 |
| Don | York Mills | 2 | 128 | 13.9 (2.2) | 3684 | 75.5 | 77.3 | 11.2 | 3.0 | 0.5 | 6.0 | 1.8 | 0.2 |
| Don | Taylor Creek | 1 | 35.7 | 7.0 (0.9) | 5721 | 90.2 | 90.7 | 5.3 | 2.3 | 0.2 | 0.4 | 1.1 | 0.0 |
| Don | German Mills | 1 | 33.2 | 7.3 (1.3) | 3300 | 82.7 | 85.7 | 5.6 | 3.0 | 0.6 | 4.0 | 1.1 | 0.0 |
| Ipswich | IP20 | 3 | 324 | 19.6 (1.1) | 332 | 24.0 | 38.9 | 32.5 | 0.8 | 23.6 | 2.0 | 0.4 | 1.8 |
| Ipswich | IP06 | 2 | 114 | 10.0 (1.7) | 494 | 37.7 | 55.3 | 20.7 | 0.6 | 21.2 | 0.4 | 0.8 | 1.1 |
| Ipswich | Sawmill Brook | 1 | 3.9 | 2.8 (0.5) | 1571 | 56.7 | 87.9 | 9.1 | 0.0 | 2.7 | 0.3 | 0.0 | 0.0 |
| Ipswich | IS163 | 1 | 3.5 | 3.6 (0.7) | 544 | 44.9 | 66.2 | 9.2 | 0.3 | 18.5 | 4.7 | 1.0 | 0.0 |

Table S2. List of item types in calibration item set.

| Item | Color | Area Range (m^2^) |
| --- | --- | --- |
| Whole Plastic Bag | Brown, Pink, White | 0.081-0.153 |
| White Plastic Bag Fragment | White | 0.045-0.12 |
| Black Plastic Bag Fragment | Black | 0.0702-0.0828 |
| Chip Bag | Black, Orange, Red, Purple, Yellow | 0.01625-0.0238 |
| Water Bottle | Clear, Red, Brown, Green | 0.0126-0.0138 |
| Bottle Cap | Black, Green, Red, White | 0.0009-0.0016 |
| Polystyrene Fragment | White | 0.0119-0.048 |
| Hard Plastic Fragment | Clear, Black | 0.014-0.0308 |

Table S3. Summary of video recorded for all floods. Events with one clear peak are listed first, followed by events with multiple peaks, then other shapes.

| Storm | Water-shed | Water-shed position | Video of pre-storm (hr) | Video of rising (hr) | Video of falling (hr) | Video of post-storm (hr) | Video of Plateau (hr) | Total Video (hr) |
| --- | --- | --- | --- | --- | --- | --- | --- | --- |
| BH2 | Chicago | 3 | 12.25 | 4.75 | 5 | 14.25 | 0 | 36.25 |
| GM1 | Don | 1 | 5.75 | 1.25 | 2 | 0 | 0 | 9 |
| GW2 | Chicago | 2 | 9.5 | 9.75 | 28.5 | 9.75 | 0 | 57.5 |
| IP062 | Ipswich | 2 | 6.75 | 8 | 0 | 0 | 0 | 14.75 |
| SR1 | Chicago | 1 | 1.5 | 4.75 | 4.5 | 15.75 | 0 | 26.5 |
| SR2 | Chicago | 1 | 0 | 4.75 | 4.25 | 9 | 0 | 18 |
| SR3 | Chicago | 1 | 5 | 9 | 22.5 | 4 | 0 | 40.5 |
| PR1 | Don | 3 | 10.25 | 0 | 8.25 | 0 | 0 | 18.5 |
| PR2 | Don | 3 | 2.25 | 4.5 | 3.25 | 0 | 0 | 10 |
| TC2 | Don | 1 | 4.75 | 2 | 2 | 0 | 0 | 8.75 |
| SB1 | Ipswich | 1 | 0 | 0 | 5.75 | 2.75 | 0 | 8.5 |
| BH1 | Chicago | 3 | 11.25 | 3.5 | 19.25 | 14 | 0 | 48 |
|  |  |  | - | - | - | - | - | - |
| BH3 | Chicago | 3 | 0 | 4.25 | 22 | 0 | 0 | 26.25 |
|  |  |  | - | - | - | - | - | - |
| GW1 | Chicago | 2 | 0 | 6.75 | 7.25 | 14.5 | 0 | 28.5 |
|  |  |  | - | - | - | - | - | - |
| TC1 | Don | 1 | 4.5 | 1.25 | 7 | 4.5 | 0 | 17.25 |
|  |  |  | - | - | - | - | - | - |
| YM1 | Don | 2 | 0 | 1.25 | 18.5 | 0 | 0 | 19.75 |
|  |  |  | - | - | - | - | - | - |
| IP201 | Ipswich | 3 | 0 | 3.5 | 0 | 0 | 8.75 | 12.25 |
| IP061 | Ipswich | 2 | 3.5 | 0 | 0 | 0 | 0 | 3.5 |

Table S4. Kruskal Wallis test results for comparing macroplastic macroplastic size (m^2^) among storm phases. Events with one clear peak are listed first, followed by events with multiple peaks, then other shapes. Watershed position: 1=tributary, 2=mid-watershed, 3=downstream. P-values <0.05 are in bold. N/A means all observed plastics were in the same phase of the hydrograph. A “-“ means there were no observed plastics.

|  |  |  | Size (m^2^) | | |  |
| --- | --- | --- | --- | --- | --- | --- |
| Site/Storm | Water-shed | Water-shed position | Test-Stat | p-value | df | |
| BH2 | Chicago | 3 | 2.33 | 0.31 | 2 | |
| GM1 | Don | 1 | 0.56 | 0.46 | 1 | |
| GW2 | Chicago | 2 | 0.82 | 0.66 | 2 | |
| IP062 | Ipswich | 2 | - | - | - | |
| SR1 | Chicago | 1 | 5.31 | 0.15 | 3 | |
| SR2 | Chicago | 1 | 0.2 | 0.66 | 1 | |
| SR3 | Chicago | 1 | 0.24 | 0.63 | 1 | |
| PR1 | Don | 3 | N/A | N/A | 0 | |
| PR2 | Don | 3 | N/A | N/A | 0 | |
| TC2 | Don | 1 | 21.48 | **<0.01** | 1 | |
| SB1 | Ipswich | 1 | - | - | - | |
| BH1 | Chicago | 3 | 2.24 | 0.52 | 3 | |
| BH3 | Chicago | 3 | N/A | N/A | 0 | |
| GW1 | Chicago | 2 | 1.35 | 0.25 | 1 | |
| TC1 | Don | 1 | 15.11 | **<0.01** | 3 | |
| YM1 | Don | 2 | 2.61 | 0.11 | 1 | |
| IP201 | Ipswich | 3 | - | - | - | |
| IP061 | Ipswich | 2 | - | - | - | |

Table S5. Total time spent, and items transported by both storms and baseflow conditions from June 1-August 31, 2022, at the Bunker Hill site in the North Branch Chicago River. Mean items per hour during pre- and post-storm hydrographs were used to estimate plastic transport during baseflow. Discharge ratio and duration of storms were used to estimate plastic transport of individual storms, which were then combined for the entire summer.

|  | Mean (Items/hr.) | Time (hours) | Proportion of Time | Items Transported (No.) | Proportion of Items |
| --- | --- | --- | --- | --- | --- |
| Baseflow | 0.32 | 1650.83 | 74.8 | 528 | 19.4 |
| Floods | - | 555.92 | 25.2 | 2193 | 80.6 |
| Total | - | 2206.75 | - | 2721 | - |

Table S6. Data for all flood events between June 1-August 31, 2022, at the Bunker Hill site in the North Branch Chicago River. Peak and Pre-flood discharge (Q) were used to calculate each flood’s discharge ratio. The discharge ratio was used to estimate macroplastic transport during each flood. We used the line of best fit from our log-scaled relationship between macroplastic transport and discharge ratio of floods to calculate these values (y = 0.854x + 1.293; y is the log-scaled number of macroplastic; x is the discharge ratio of the flood).

| Start | Pre-flood Q | Peak  Q | End | Time in flood | Baseflow time between floods | Q ratio | Log (Q ratio) | y | No. items during flood 10^y^ |
| --- | --- | --- | --- | --- | --- | --- | --- | --- | --- |
| 6/1/2022 0:00 | 1.38 | 3.28 | 6/1/2022 8:45 | 8:45:00 | 171:30:00 | 2.38 | 0.38 | 1.61 | 41 |
| 6/8/2022 12:15 | 1.76 | 8.47 | 6/10/2022 12:30 | 48:15:00 | 77:00:00 | 4.81 | 0.68 | 1.88 | 75 |
| 6/13/2022 17:30 | 1.1 | 9.26 | 6/15/2022 0:40 | 31:10:00 | 23:20:00 | 8.42 | 0.93 | 2.08 | 121 |
| 6/16/2022 0:00 | 1.37 | 5.04 | 6/16/2022 5:15 | 5:15:00 | 450:00:00 | 3.68 | 0.57 | 1.78 | 60 |
| 7/4/2022 23:15 | 0.27 | 15.4 | 7/6/2022 0:30 | 25:15:00 | 0:00:00 | 57.04 | 1.76 | 2.79 | 621 |
| 7/6/2022 0:30 | 4.5 | 13 | 7/7/2022 20:30 | 44:00:00 | 106:45:00 | 2.89 | 0.46 | 1.69 | 49 |
| 7/12/2022 7:15 | 0.48 | 1.211 | 7/13/2022 5:00 | 21:45:00 | 50:00:00 | 2.52 | 0.40 | 1.64 | 43 |
| 7/15/2022 7:00 | 0.5 | 7.7 | 7/15/2022 16:00 | 9:00:00 | 180:15:00 | 15.40 | 1.19 | 2.31 | 203 |
| 7/23/2022 4:15 | 0.34 | 19.68 | 7/27/2022 13:15 | 105:00:00 | 0:00:00 | 57.88 | 1.76 | 2.80 | 628 |
| 7/27/2022 13:15 | 10.96 | 18.89 | 7/30/2022 14:00 | 72:45:00 | 109:15:00 | 1.72 | 0.24 | 1.49 | 31 |
| 8/4/2022 3:15 | 0.89 | 1.31 | 8/4/2022 14:45 | 11:30:00 | 80:00:00 | 1.47 | 0.17 | 1.44 | 27 |
| 8/7/2022 22:45 | 1.23 | 2.55 | 8/10/2022 5:15 | 54:30:00 | 236:45:00 | 2.07 | 0.32 | 1.56 | 37 |
| 8/20/2022 2:00 | 0.52 | 3.31 | 8/21/2022 6:45 | 28:45:00 | 105:30:00 | 6.37 | 0.80 | 1.98 | 95 |
| 8/25/2022 16:15 | 0.99 | 3.97 | 8/26/2022 5:30 | 13:15:00 | 60:30:00 | 4.01 | 0.60 | 1.81 | 64 |
| 8/28/2022 18:00 | 1.27 | 8.26 | 8/31/2022 22:45 | 76:45:00 | 0:00:00 | 6.50 | 0.81 | 1.99 | 97 |
|  |  |  |  |  |  |  |  | *Total* | 2193 |


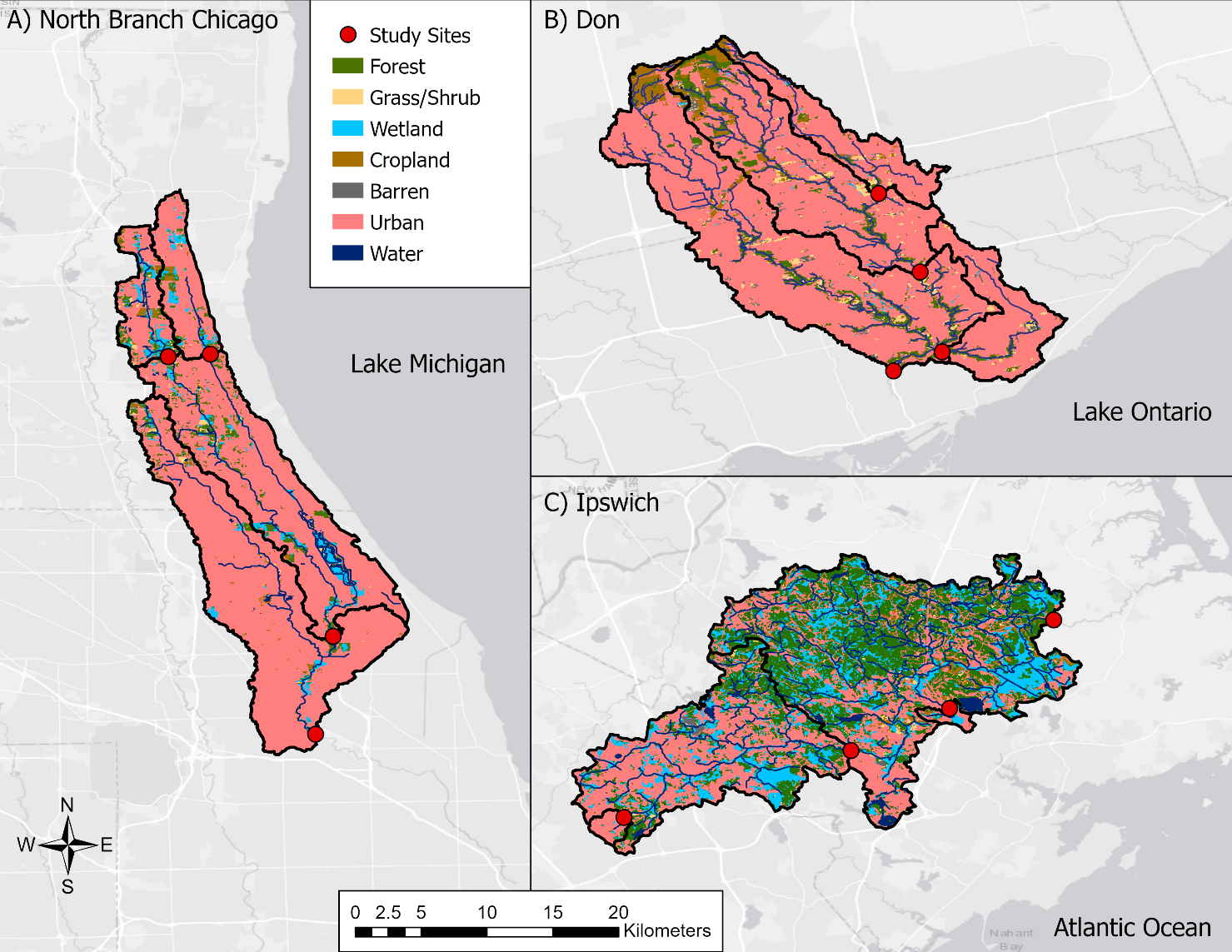


Figure S1. Three study watersheds: North Branch Chicago River (A), Don River (B), and Ipswich River (C). Colors represent different forms of land use for each watershed. Study sites are shown as red circles.


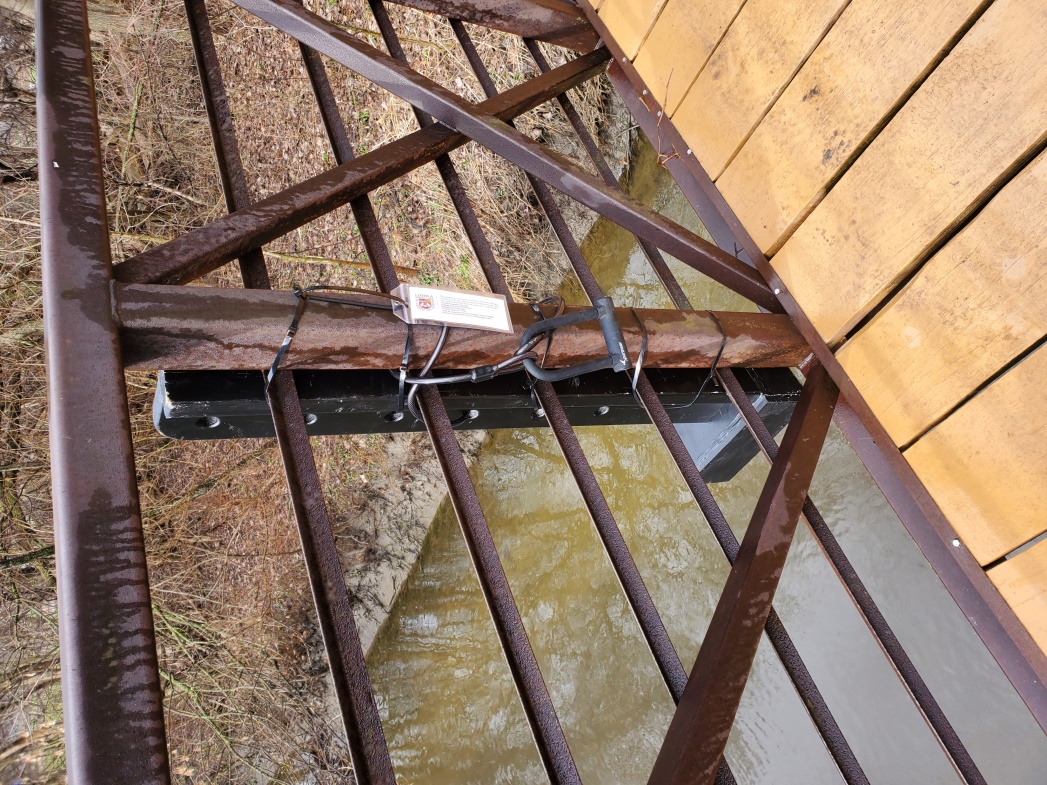


Figure S2. The camera mount attached to a pedestrian bridge. The camera was attached under the bottom portion of the mount and recorded the surface of the stream.


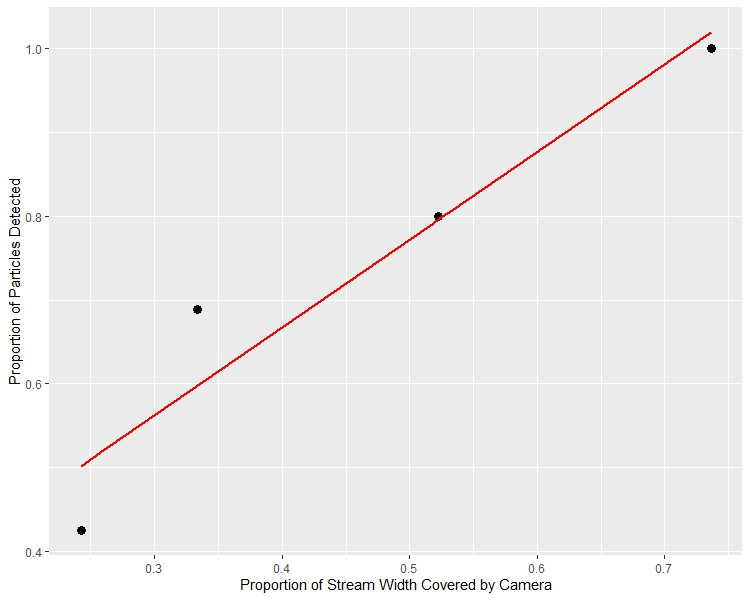


Figure S3. Macroplastic calibration analysis for video camera particle detection, completed at four sites in the North Branch Chicago River. R^2^=0.915, p=0.044, line of best fit: y= 1.048x + 0.2479.


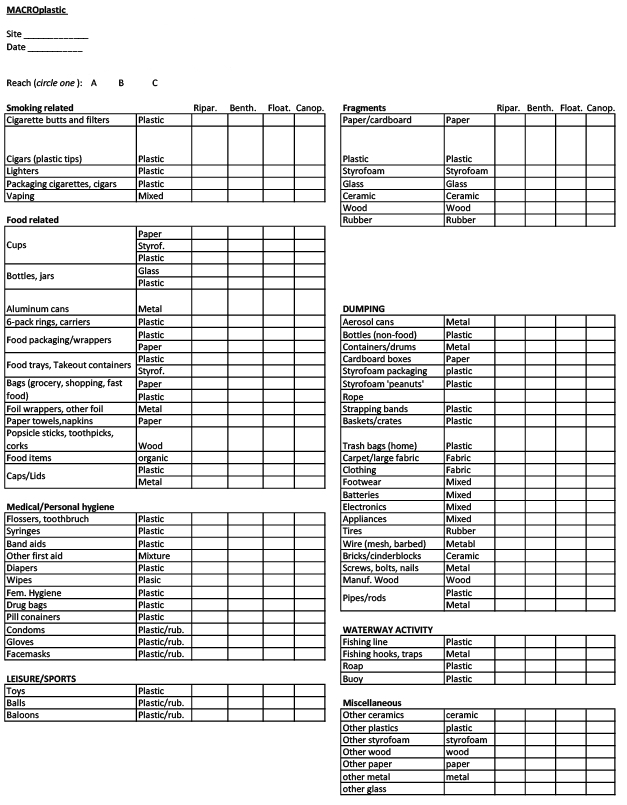


Figure S4. Datasheet used to categorize all collected anthropogenic litter (AL). The first column is the function of the collected item, the second column is the general material type of the AL, and columns 3-6 are the habitats the AL was found in.


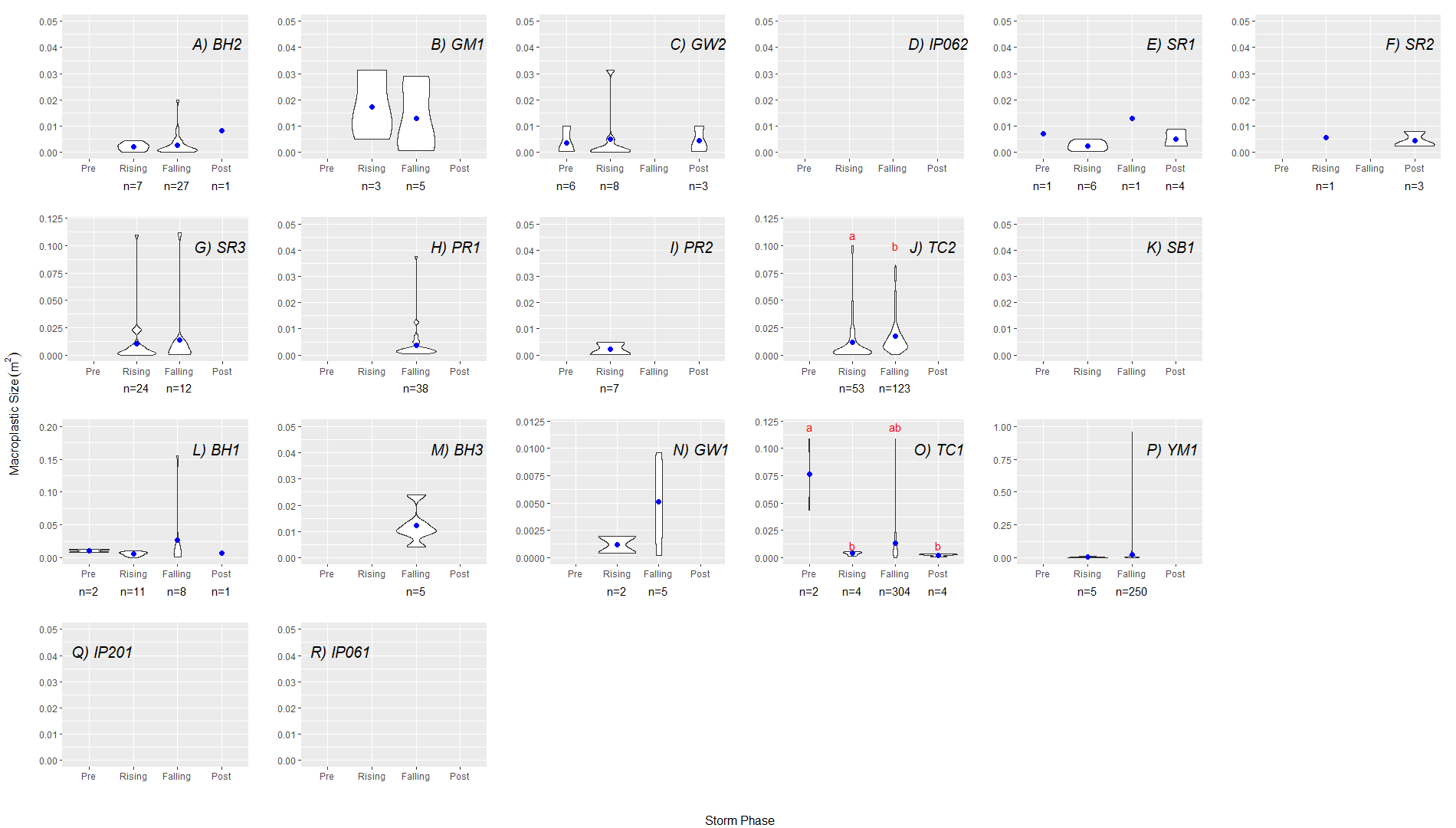


Figure S5. Violin plots showing macroplastic size by storm phase (points = mean). The total number of items reported for each phase is shown below the plots. Small letters represent differences (Dunn’s multiple comparison test) after a significant Kruskal-Wallis test among phases.


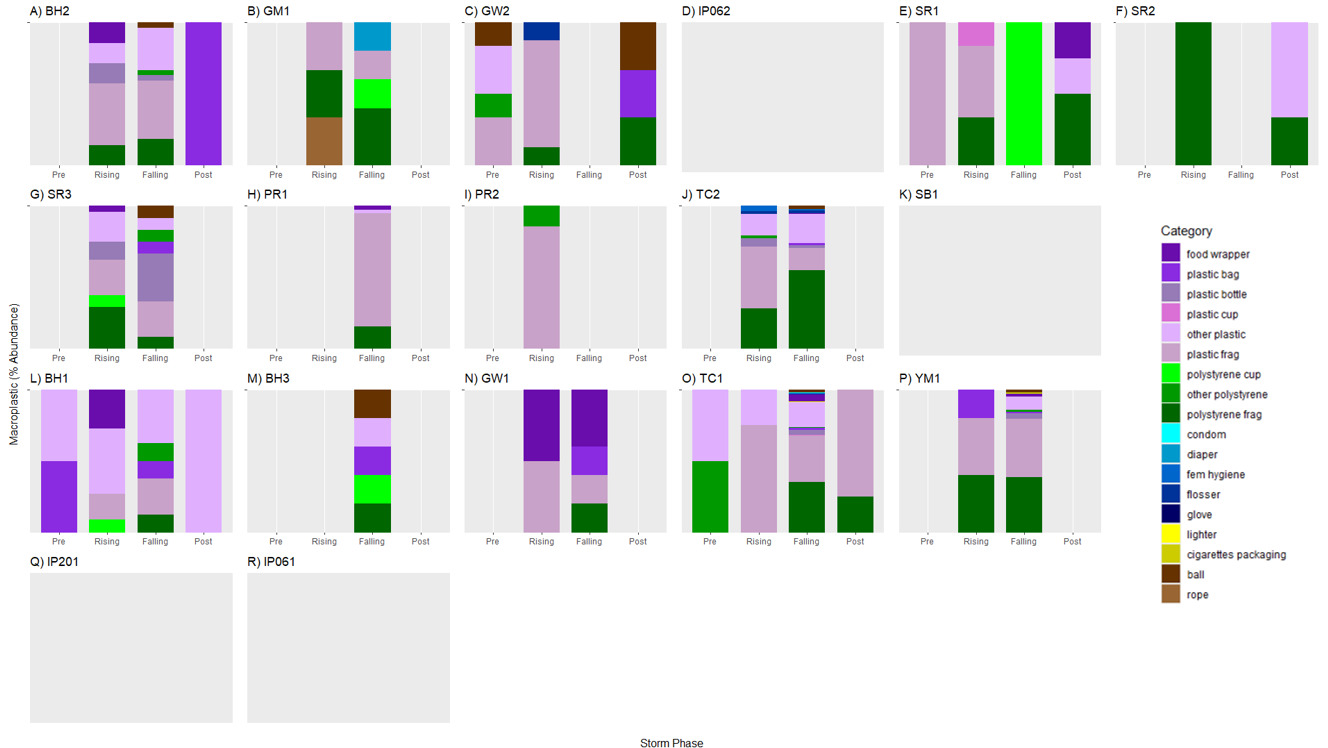


Figure S6. The relative abundance of macroplastic categories detected by storm phase. Shades of red represent non-polystyrene plastics, shades of green represent expanded polystyrene plastics, shades of blue represent hygiene products, and the other colors represent miscellaneous plastics.


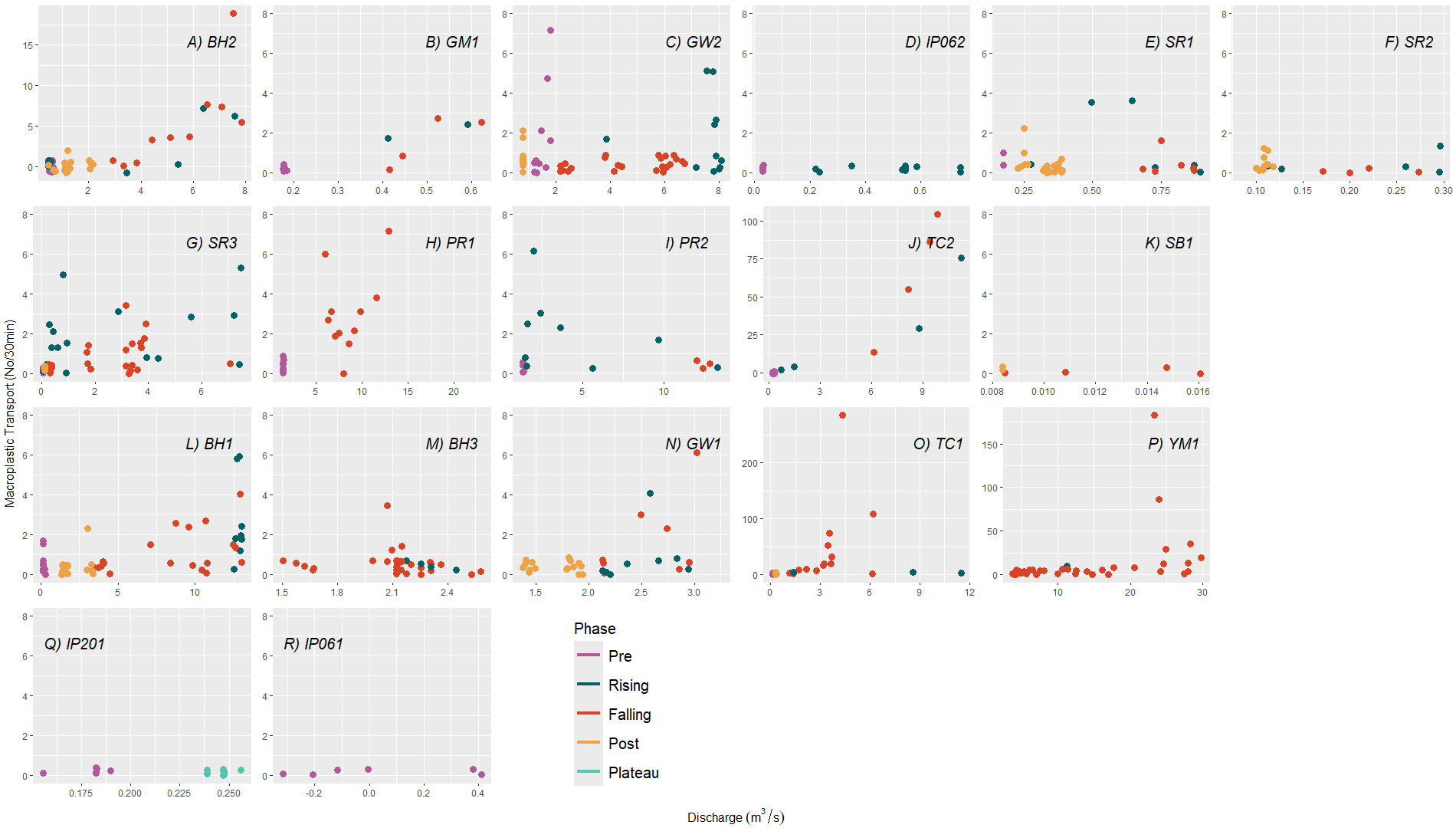


Figure S7. Macroplastic transport rate by stream discharge, where the colors show storm phase. Jitter was added to the plots for a better visualization.


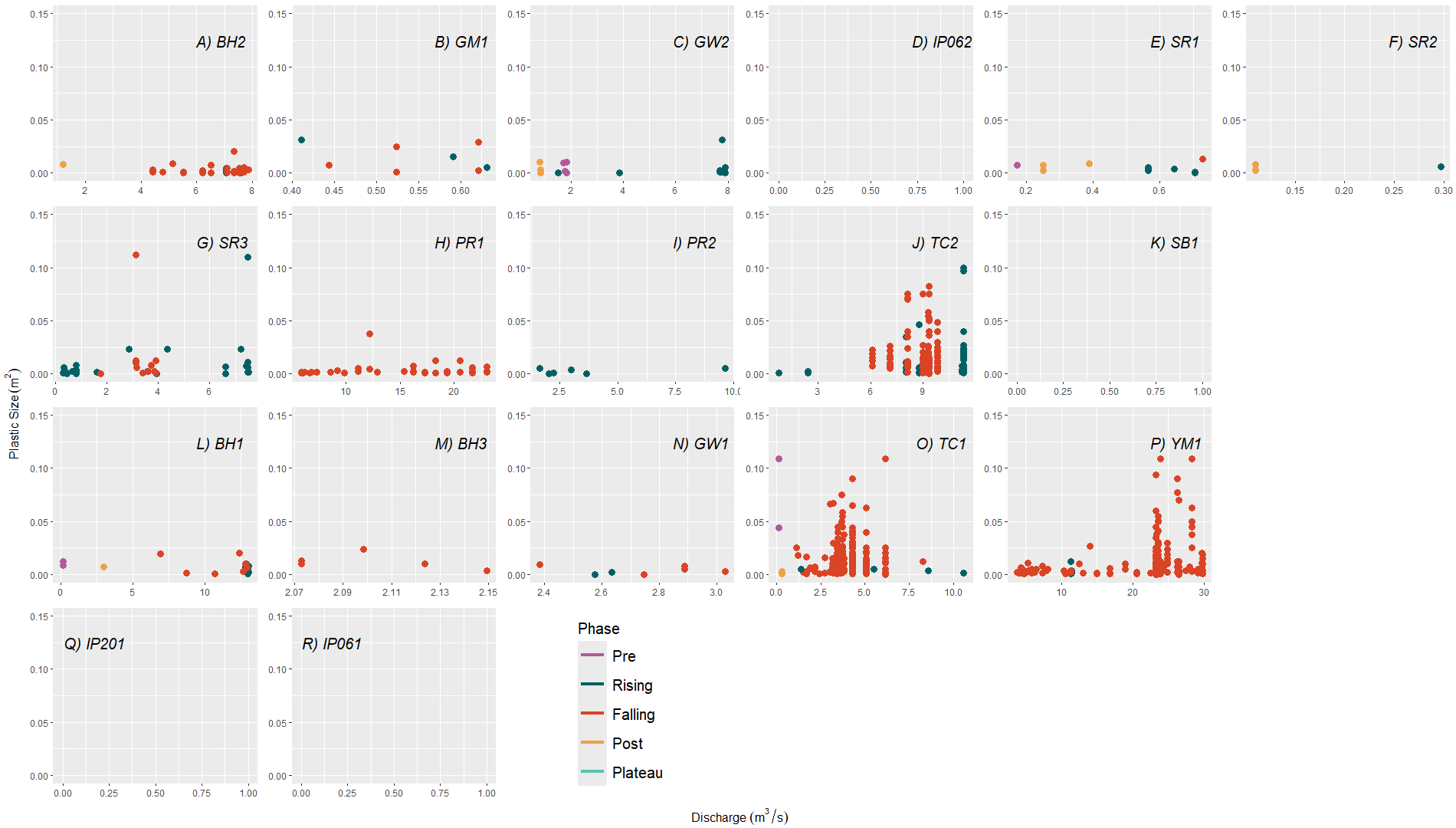


Figure S8. Size of macroplastics by discharge, where point color represents storm phase.


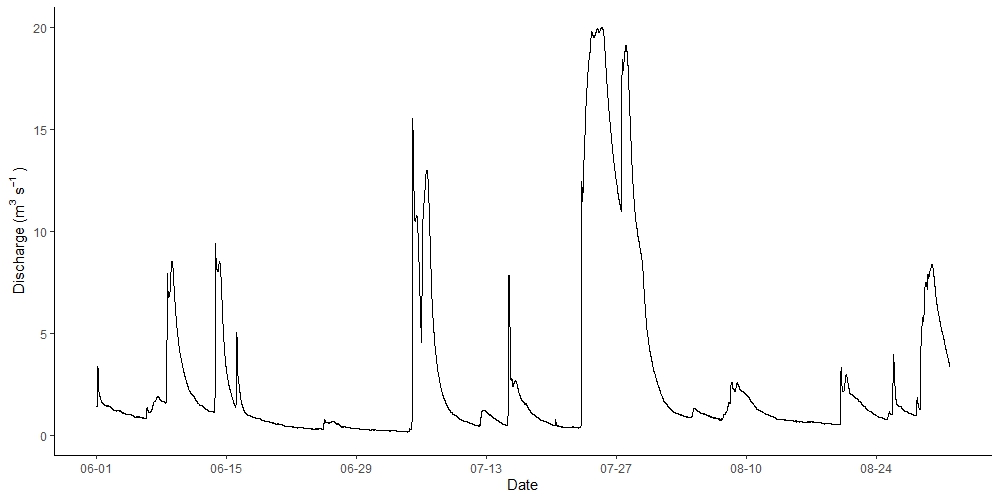


Figure S9. Hydrograph for June 1^st^- August 31^st^, 2022, at Bunker Hill. Date as “month/day” is shown on the x-axis.
